# The Effects of Cariprazine and Aripiprazole on PCP-Induced Deficits on Attention Assessed in the 5-Choice Serial Reaction Time Task

**DOI:** 10.1007/s00213-018-4857-0

**Published:** 2018-02-22

**Authors:** Samuel A. Barnes, Jared W. Young, Athina Markou, Nika Adham, István Gyertyán, Béla Kiss

**Affiliations:** 1 Department of Psychiatry, School of Medicine, University of California San Diego, 9500 Gilman Drive, M/C 0603, Room BSB2202, La Jolla, CA 92093 USA; 2 Allergan, Jersey City, NJ USA; 3 MTA-SE NAP B Cognitive Translational Behavioral Pharmacology Group, Department of Pharmacology and Pharmacotherapy, Semmelweis University, Budapest, Hungary; 4 Institute of Cognitive Neuroscience and Psychology, Research Center for Natural Sciences, MTA, Budapest, Hungary; 5 Gedeon Richter Plc, Budapest, Hungary

**Keywords:** Cariprazine, 5-CSRTT, Aripiprazole, Schizophrenia, Cognition, PCP

## Abstract

**Rationale:**

Attentional processing deficits are a core feature of schizophrenia, likely contributing to the persistent functional and occupational disability observed in patients with schizophrenia. The pathophysiology of schizophrenia is hypothesized to involve dysregulation of NMDA receptor-mediated glutamate transmission, contributing to disruptions in normal dopamine transmission. Preclinical investigations often use NMDA receptor antagonists, such as phencyclidine (PCP), to induce cognitive disruptions relevant to schizophrenia. We sought to test the ability of partial dopamine D_2_/D_3_ agonists, cariprazine and aripiprazole, to attenuate PCP-induced deficits in attentional performance.

**Objectives:**

The objective of this study is to determine whether systemic administration of cariprazine or aripiprazole attenuated 5-choice serial reaction time task (5-CSRTT) deficits induced by repeated exposure to PCP.

**Methods:**

We utilized a repeated PCP-treatment regimen (2 mg/kg, subcutaneous [s.c.], once daily for 5 days) in rats to induce deficits in the 5-CSRTT. Rats were pre-treated with cariprazine (0.03, 0.1, or 0.3 mg/kg, oral [p.o.]) or aripiprazole (1, 3, or 10 mg/kg, p.o.) to determine whether they prevented PCP-induced deficits in the 5-CSRTT performance.

**Results:**

PCP treatment increased inappropriate responding in the 5-CSRTT, elevating incorrect, premature, and timeout responses. Cariprazine treatment reduced PCP-induced increases in inappropriate responding. However, at higher doses, cariprazine produced non-specific response suppression, confounding interpretation of the attenuated PCP-induced deficits. Aripiprazole treatment also attenuated PCP-induced deficits; however, unlike cariprazine treatment, aripiprazole reduced correct responding and increased omissions.

**Conclusions:**

Cariprazine and aripiprazole both demonstrated potential in attenuating PCP-induced deficits in the 5-CSRTT performance. While both compounds produced non-specific response suppression, these effects were absent when 0.03 mg/kg cariprazine was administered.

## Introduction

Schizophrenia is a chronic disorder associated with psychotic disturbances, negative symptoms, and cognitive dysfunction. Cognitive deficits are a prominent feature of schizophrenia. Indeed, cognitive impairments are evident before the onset of psychotic outbreaks, persist despite therapeutic intervention, and appear to be a stable feature of the illness. As cognitive impairments are largely unresponsive to currently available medications and are thought to significantly contribute to the functional disability associated with the disorder, the identification of novel and efficacious therapeutic strategies is essential.

The National Institute of Mental Health’s Measurement and Treatment Research to Improve Cognition in Schizophrenia (MATRICS) initiative identified several cognitive domains that are disrupted in patients with schizophrenia, including attention and vigilance (Kern et al. 2004; Young and Geyer 2015). Attention is a cognitive process that allows an individual to detect, select, and process sensory stimuli (Maunsell and Treue 2006). Impairments in attention have been associated with schizophrenia since the earliest descriptions of the disorder (Kraepelin 1921). Furthermore, attentional processing is suggested to form an underlying basis of several higher-order cognitive processes (Riedel et al. 2006), many of which were also implicated by the MATRICS initiative as being disrupted in schizophrenia (Kern et al. 2004). Thus, attentional deficits in schizophrenia may contribute to the spectrum of cognitive dysfunction observed in patients and the associated functional disabilities. Identifying the mechanism(s) that contribute to attentional deficits, and subsequent therapeutic strategies to improve attentional functioning, may be a key to improving the patients’ quality of life.

*N*-methyl-d-aspartate (NMDA) glutamate receptor hypofunction is hypothesized to contribute to the pathophysiology of schizophrenia, as NMDA receptor antagonists can induce schizophrenia-like symptoms in otherwise healthy individuals that recapitulate aspects of positive, negative, and cognitive symptomatology (Corlett et al. 2007; Krystal et al. 1994; Pomarol-Clotet et al. 2006). Indeed, it has recently been demonstrated that patients with schizophrenia may exhibit alterations in the expression of key functional subunits, potentially leading to endogenous NMDA receptor dysfunction in schizophrenia (Weickert et al. 2013). In addition to glutamate transmission, cortical hypofunction and sub-cortical hyperfunction of dopamine transmission are reported in patients with schizophrenia (Howes et al. 2015). Importantly, dopamine transmission is implicated in attentional performance (Barnes et al. 2012; Boulougouris and Tsaltas 2008; Carli and Invernizzi 2014; Granon et al. 2000; Nieoullon 2002), and augmenting dopamine transmission has been suggested as a potential target in the development of procognitive therapeutics for the treatment of patients with schizophrenia (Gray and Roth 2007; Ibrahim and Tamminga 2011). Furthermore, alterations in glutamate transmission can impact normal dopaminergic transmission, suggesting that hypotheses that involve these neurotransmitter systems and schizophrenia pathogenesis may not be mutually exclusive. Indeed, NMDA receptor antagonists have been used in experimental animals to induce deficits in cognitive processing relevant to schizophrenia (Grayson et al. 2015; Jentsch and Roth 1999; Neill et al. 2010; Neill et al. 2014; Pratt et al. 2008). Moreover, NMDA receptor antagonism produces alterations in dopamine transmission (Adams et al. 2002; Kapur and Seeman 2002). Hence, the use of experimental animal models that utilize NMDA receptor antagonists to induce deficits in attentional processing, coupled with dopaminergic manipulations to improve attention, may provide insights into the mechanism(s) that contribute to attention deficits in patients with schizophrenia.

The 5-choice serial reaction time task (5-CSRTT) is a well-validated and widely used behavioral procedure that tests attentional deficits in experimental rodents (Lustig et al. 2012), and the neural substrates involved in task performance have been well described (Chudasama and Robbins 2004; Robbins 2002). Previous investigations revealed that the administration of NMDA receptor antagonists (i.e., phencyclidine [PCP] or ketamine) to experimental animals impairs attentional processing (Amitai and Markou 2009, 2010; Amitai et al. 2007; Barnes et al. 2014; Nikiforuk and Popik 2014; Thomson et al. 2011). However, acute administration of PCP disrupts the 5-CSRTT performance in a generalized manner that is difficult to interpret as a specific cognitive impairment (Amitai and Markou 2010; Carli and Invernizzi 2014). Therefore, researchers find it advantageous to use a NMDA antagonist-treatment regimen that allows testing after a washout period or after repeated exposure to allow tolerance to the non-specific disruptions to develop (Barnes et al. 2014; Barnes et al. 2016; Nikiforuk and Popik 2014; Thomson et al. 2011). For instance, we have previously shown that a repeated PCP-treatment regimen induces cognitive-specific deficits in 5-CSRTT performance (Amitai and Markou 2009, 2010; Amitai et al. 2007). Deficits in 5-CSRTT performance were largely attributed to an increase in incorrect responses, omissions, and premature responses. These findings suggest that aberrant glutamatergic transmission may contribute to the attentional deficits evident in patients with schizophrenia. Furthermore, PCP-induced deficits in 5-CSRTT performance were attenuated by clozapine (Amitai et al. 2007) but not quetiapine treatment (Amitai and Markou 2009). Interestingly, clozapine displays a greater receptor occupancy than quetiapine for striatal dopamine receptors (Tauscher et al. 2004). This occupancy difference may account for the differing efficacy in ameliorating PCP-induced deficits. Using a pharmacological strategy that attenuates PCP-induced deficits in attentional performance by augmenting dopamine transmission may represent an effective therapeutic strategy for patients with schizophrenia.

Cariprazine is FDA approved to treat adults with schizophrenia and manic or mixed episodes associated with bipolar disorder. Cariprazine is a dopamine D_3_/D_2_ receptor partial agonist that preferentially binds to the dopamine D_3_ receptor (Kiss et al. 2010). It has shown procognitive and prosocial effects in rodents, improving PCP-induced deficits in executive functioning, working memory, recognition memory, and social interaction (Neill et al. 2016; Zimnisky et al. 2013). Interestingly, dopamine D_3_ receptor expression is most abundant in mesolimbic regions (Kiss et al. 2010) that may be involved in attentional processing (Feja et al. 2014; St. Peters et al. 2011). Cariprazine may therefore modulate 5-CSRTT performance and attenuate PCP-induced attentional deficits by altering mesolimbic dopamine D_3_ receptor activity. Aripiprazole is an FDA approved compound for the treatment of schizophrenia that has D_2_ receptor partial agonist activity and a similar pharmacological profile to cariprazine (Kiss et al. 2010). However, unlike cariprazine, it shows very low occupancy of D_3_ receptors within its antipsychotic-like effective doses that occupy D_2_ receptors to a high extent (≥ 80%) (Gyertyán et al. 2011). Interestingly, a disruption of the 5-CSRTT performance induced by the infusion of the NMDA receptor antagonist 3-(*R*)-2-carboxypiperazin-4-propyl-1-phosphonic acid (CPP) into the medial prefrontal cortex was attenuated by aripiprazole (Carli et al. 2011). Aripiprazole has also shown efficacy in the treatment of cognitive and negative symptoms in experimental animal models (Nagai et al. 2009; Wilson and Koenig 2014). Therefore, the aim of the present study was to identify whether systemic administration of cariprazine or aripiprazole attenuated 5-CSRTT deficits induced by repeated exposure to PCP and to explore eventual differences between these compounds in this model.

## Experimental procedures

### Animals

Male Wistar rats (weighing approximately 225 g; *n* = 96) were purchased from Charles River Laboratories (Raleigh, NC) and housed two per cage in a climate-controlled room on a 12 h/12 h reversed light-dark cycle (lights on at 6:00 p.m.); all behavioral testing was conducted in the animals’ dark cycle. No environmental enrichment was provided. Food and water were available ad libitum until behavioral testing began, during which time access to food was restricted. All experiments were conducted in accordance with the guidelines from the National Institutes of Health and the Association for the Assessment and Accreditation of Laboratory Animal Care and were approved by the University of California, San Diego Institutional Animal Care and Use Committee.

### Drugs

Phencyclidine hydrochloride (PCP) (Sigma Aldrich, MO) was dissolved in 0.9% saline and administered by a subcutaneous (s.c.) injection of a volume of 2 ml/kg and a concentration of 2 mg/kg. Aripiprazole (Forest Laboratories, NY) was suspended in 2% Tween 80 in distilled H_2_O and administered by oral (p.o.) gavage (1, 3, or 10 mg/kg). Cariprazine HCl (Forest Laboratories, NY) was also dissolved in 2% Tween 80 and administered p.o. (0.03, 0.1, or 0.3 mg/kg). Drugs were administered according to the treatment regimen described as follows by an experimenter blind to all treatment groups throughout testing.

### Behavioral apparatus

Training and testing were conducted in operant conditioning boxes (Med Associates, St Albans, VT). Each box was enclosed in a wooden sound-attenuating chamber and contained a curved rear wall with nine response apertures, each containing a photobeam at the entrance of each aperture to detect nose pokes and a 3-W yellow light to provide a visual stimulus. Metal inserts blocked four response apertures, leaving holes 1, 3, 5, 7, and 9 free for exploration. On the opposite wall, a food magazine connected to a food hopper enabled the delivery of food pellets (45 mg rodent pellet, Test Diet 5TUM, Richmond, IN), which was signaled by the illumination of a 3-W bulb in the food magazine. A photobeam detected head entries into the food magazine. Each box was controlled by a PC running the MedPC software (Med Associates).

### Training

Animals were food restricted to 85% of their free-feeding body weight, although water access was available ad libitum. Food restriction continued throughout training and testing. Training was conducted as previously described (Amitai et al. 2007). Briefly, training began by habituating animals to the 5-CSRTT chambers for 20 min for 2 days. This initial habituation was followed by two 20-min sessions in which a food pellet was non-contingently delivered every 20 s. During these initial training sessions, each response aperture was baited with food pellets to encourage exploration. Response training began with all response apertures illuminated in a session lasting 30 min. Response in any aperture delivered a reward pellet; criteria to move onto the next stage was > 70 completed trials.

Animals were then trained in the 5-CSRTT procedure; one response aperture was illuminated for each trial. A response in the illuminated aperture resulted in the delivery of a reward pellet. A response in any aperture that was not illuminated was deemed an incorrect response and resulted in a 5-s timeout period (house light extinguished and no food reward delivered). Failure to respond (omission), responding during the inter-trial interval (ITI) before the visual stimulus was presented (premature response), or repeated responses after a correct response (perseverative response) also resulted in a timeout. Responses made during the timeout period restarted the 5-s timeout period and were recorded as a timeout response. These measures are summarized in Table 1. The stimulus duration was initially 30 s and progressively decreased (20, 10, 5, 2.5, 1.5 s) as individual animals reached the predetermined criteria (> 70 trials completed, > 70% accuracy, and < 20% omissions) until animals reached the target stimulus duration of 1 s. Each session lasted 30 min or when 100 trials had been completed, whichever occurred first.

**Table 1:** Description of the behavioral measures assessed in the 5-CSRTT

Measure	Description
Accuracy	Correct responses / (correct + incorrect) × 100
Percent correct	Correct response / (correct + incorrect + omissions) × 100
Percent incorrect	Incorrect response / (correct + incorrect + omissions) × 100
Percent omission	Omitted responses / (correct + incorrect + omissions) × 100
Premature	Response made during ITI prior to stimulus onset
Perseverative	Initial inappropriate repeat response following a correct response
Timeout	Subsequent inappropriate repeat responses made during the timeout period
Correct latency	Time taken to make a correct response
Reward latency	Time take to collect the food reward

Further information describing these measures can be found in Amitai and Markou (2010)

### Experimental design

**Fig. 1:**
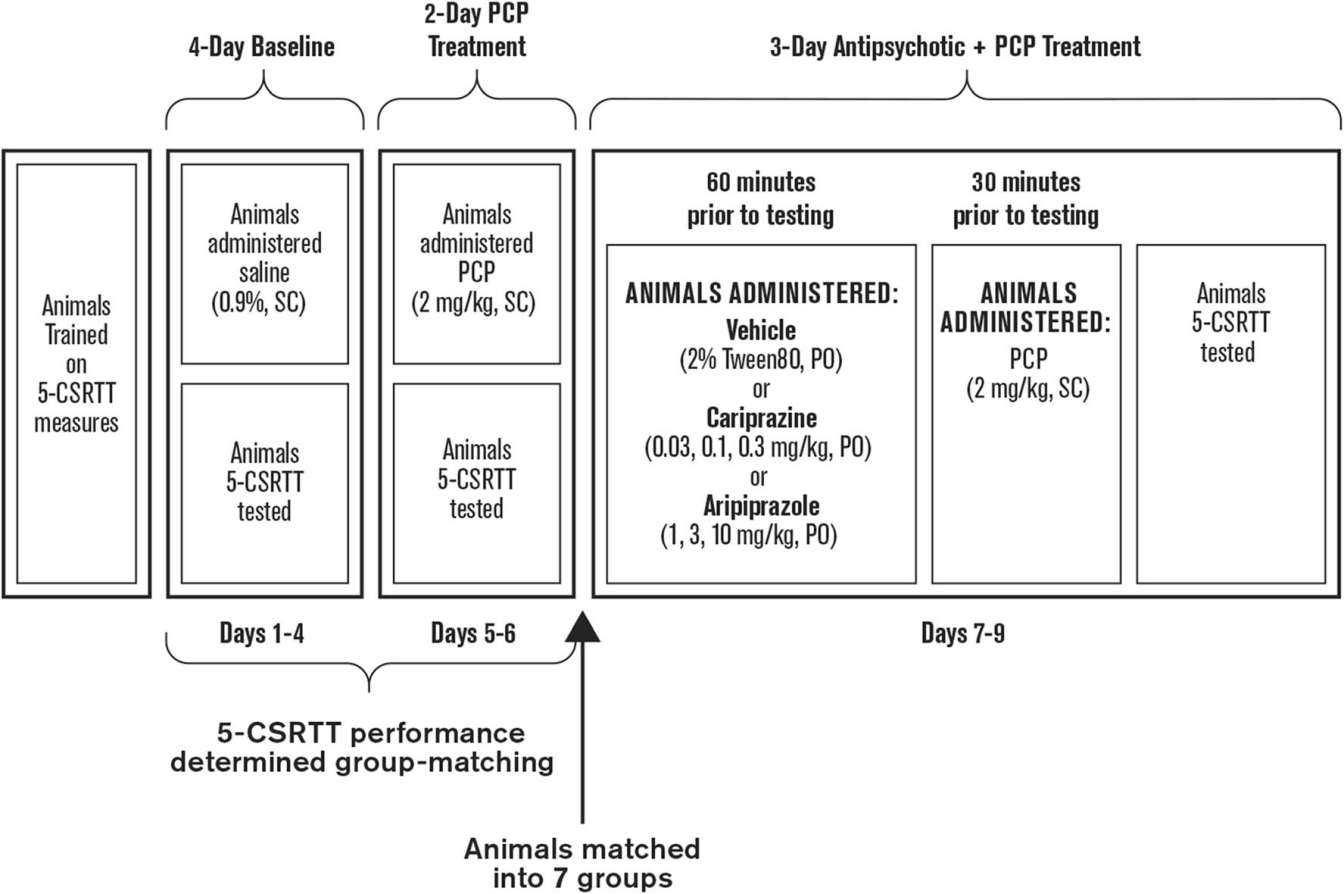
Experimental study design. 5-CSRTT 5-choice serial reaction time test, PCP phencyclidine, PO oral gavage, SC subcutaneous

Once trained, animals were split into seven groups (Fig. 1). All animals were administered saline (0.9%, s.c.) 30 min before 5-CSRTT testing for 4 days. Then, all animals were administered PCP (2 mg/kg, s.c.) for 5 consecutive days. Using the 4 days of saline treatment and the initial 2 days of PCP treatment, the performance of the seven treatment groups was balanced to minimize any difference in behavioral performance (in the absence and presence of PCP) between groups before aripiprazole or cariprazine were administered. Group-matching was based on response accuracy, premature responding, percent correct, percent omissions, correct latency, and reward latency, as previously described (Amitai et al. 2007). After group-matching, the animals were administered vehicle (2% Tween 80, p.o.), aripiprazole (1, 3, or 10 mg/kg, p.o.), or cariprazine (0.03, 0.1, or 0.3 mg/kg, p.o.) 60 min before 5-CSRTT testing. After 30 min, all animals were administered PCP (2 mg/kg, s.c.), and 5-CSRTT testing was initiated after a 30-min pre-treatment time.

### Data analysis

The baseline performance, consisting of 4 days of saline treatment, and the final 3 days of the PCP + cariprazine or aripiprazole treatment were averaged. Data were analyzed by two-way repeated measures ANOVA (day as repeated measures and treatment as the between-subject variable), followed by a Fisher’s LSD post hoc test where appropriate. The a priori hypothesis was that repeated PCP treatment would disrupt incorrect responding, according to previous observations (Amitai and Markou 2009; Amitai et al. 2007). Sample size was selected based on prior experience from our lab with this treatment regimen and behavioral procedure. As a result, no formal power analysis was performed. Data were expressed as means ± SEM, analyzed in Statsoft Statistica v8, and displayed in GraphPad Prism v5.

## Results

**Fig. 2:**
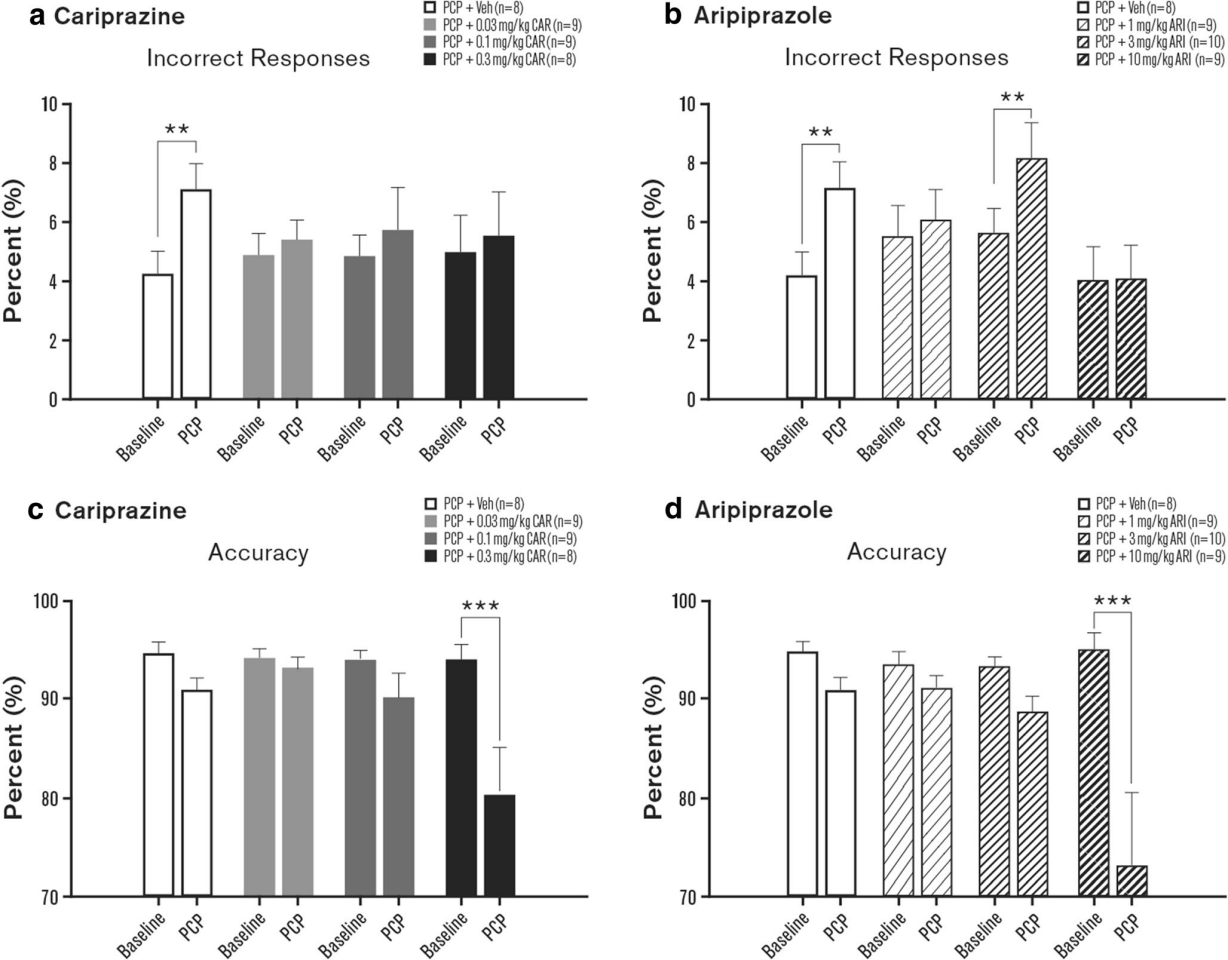
Effects of cariprazine and aripiprazole on PCP-induced alterations in attentional processing: incorrect responses (**a**, **b**) and response accuracy (**c**, **d**). Measures are shown as means ± SEM; *p* values are based on ANOVA planned comparisons (cariprazine; incorrect responses) or post hoc Fisher least significant difference tests (cariprazine; response accuracy, and aripiprazole). **p < 0.01 compared to baseline. ARI aripiprazole, CAR cariprazine, PCP phencyclidine, Veh vehicle

Percent incorrect responses, percent correct responses, accuracy, and percent omissions were evaluated in rats. Although the day × treatment interaction for percent incorrect responding failed to reach significance [*F*_(3, 30)_ = 1.75, *p* = 0.18], preplanned comparisons demonstrated that PCP alone significantly increased incorrect responding compared to baseline (*p* < 0.01; Fig. 2a, Table 2). This increase in incorrect responding was not observed in cariprazine-treated (0.01–0.3 mg/kg) animals (Fig. 2a, Table 2). Significant interactions were evident for percent incorrect responses in the aripiprazole treatment experiments [*F*_(3, 32)_ = 3.32, *p* < 0.05]. PCP alone increased the percentage of incorrect responses (*p* < 0.01), but this effect was attenuated when aripiprazole (1 or 10 mg/kg) was administered (Fig. 2b, Table 2). At 3 mg/kg, aripiprazole was not effective in attenuating the PCP-induced increase in incorrect responses (*p* < 0.01; Fig. 2b, Table 2).

**Table 2:** 5-CSRTT performance

Measure		PCP + Veh	PCP + cariprazine			PCP + aripiprazole		
			0.03 mg/kg	0.1 mg/kg	0.3 mg/kg	1 mg/kg	3 mg/kg	10 mg/kg
Incorrect responses (%)	Baseline	4.3 (0.8)	4.9 (0.8)	4.9 (0.7)	5.0 (1.2)	5.8 (1.1)	5.6 (0.8)	4.0 (1.2)
	Treatment	7.1 (0.9)*	5.4 (0.7)	5.7 (1.4)	5.5 (1.5)	6.0 (1.1)	8.2 (1.2)*	4.1 (1.2)
Correct reponses (%)	Baseline	78.4 (3.0)	79.2 (3.3)	75.6 (2.1)	79.2 (3.3)	79.4 (2.5)	79.2 (2.3)	80.3 (3.2)
	Treatment	71.4 (3.0)	76.6 (3.4)	51.6 (2.6)*	36.4 (6.0)*	65.2 (4.9)*	66.9 (3.0)*	38.7 (5.2)*
Accuracy (%)	Baseline	94.7 (1.1)	94.1 (1.0)	94.0 (0.9)	93.9 (1.6)	93.4 (1.3)	93.2 (1.1)	95.0 (1.7)
	Treatment	90.8 (1.3)	93.1 (1.0)	90.1 (2.4)	80.3 (4.9)*	91.0 (1.3)	88.6 (1.7)	73.0 (7.5)*
Omissions (%)	Baseline	17.3(2.6)	16.0 (3.1)	19.5 (2.0)	15.7 (2.9)	15.0 (2.3)	15.2 (1.7)	15.8 (2.4)
	Treatment	21.5 (2.1)	18.0 (3.1)	42.6 (2.8)*	58.2 (6.8)*	28.8 (5.1)*	24.9 (2.5)*	57.2 (5.7)*
Trials completed, *n*	Baseline	99.1 (0.6)	99.1 (0.6)	98.9 (0.7)	96.6 (2.5)	99.0 (0.5)	99.4 (0.4)	99.1 (0.9)
	Treatment	89.1 (5.6)	97.7 (1.0)	79.2 (5.9)*	49.1 (9.8)*	86.6 (6.3)	94.6 (2.5)	56.1 (9.6)*
Premature responses, *n*	Baseline	6.7 (1.4)	10.3 (1.1)	8.3 (1.7)	9.7 (2.6)	11.0 (3.1)	9.3 (1.7)	6.8 (1.2)
	Treatment	12.8 (3.3)*	10.1 (1.8)	3.9 (1.4)	2.5 (1.2)*	9.4 (2.4)	8.2 (2.4)	2.7 (0.9)
Timeout responses, *n*	Baseline	6.8 (1.1)	7.5 (1.4)	5.3 (1.0)	7.8 (1.6)	7.0 (2.3)	8.8 (1.6)	6.2 (1.3)
	Treatment	12.9 (3.2)*	9.4 (1.3)	4.6 (1.6)	4.3 (1.5)	6.6 (1.3)	14.0 (2.7)*	5.0 (1.6)
Correct latency (s)	Baseline	0.8 (0.0)	0.8 (0.0)	0.8 (0.0)	0.8 (0.0)	0.8 (0.0)	0.9 (0.0)	0.8 (0.0)
	Treatment	0.8 (0.0)	0.8 (0.0)	1.0 (0.0)	1.0 (0.1)	0.9 (0.1)	0.9 (0.0)	0.9 (0.1)
Reward latency (s)	Baseline	1.8 (0.1)	1.7 (0.2)	2.1 (0.2)	2.1 (0.3)	1.8 (0.1)	2.0 (0.2)	1.7 (0.2)
	Treatment	1.6 (0.2)	1.5 (0.1)	2.5 (0.3)	1.8 (0.3)	1.7 (0.1)	2.0 (0.2)	1.5 (0.2)
Perseverative responses, *n*	Baseline	6.5 (1.0)	5.8 (1.2)	6.8 (1.4)	9.8 (1.8)	7.2 (0.7)	8.7 (2.1)	7.0 (0.8)
	Treatment	7.3 (1.9)	7.2 (0.8)	5.6 (1.2)	2.8 (1.2)*	8.1 (2.3)	8.0 (1.2)	4.7 (1.6)

Measures are shown as mean ± SEM

*5-CSRTT* 5-choice serial reaction time task, *PCP* phencyclidine, *Veh* vehicle

*p < 0.05

For response accuracy, significant interactions were evident with cariprazine [*F*_(3, 30)_ = 3.81, *p* < 0.05] and aripiprazole [*F*_(3, 32)_ = 5.51, *p* < 0.01]. Accuracy was significantly reduced by PCP and 0.3 mg/kg cariprazine (*p* < 0.001; Table 2; Fig. 2c) and by PCP and 10 mg/kg aripiprazole (*p* < 0.001; Fig. 2d), but not by PCP alone. Similarly, the analysis of the percentage of correct responses also resulted in significant interactions for cariprazine [*F*_(3, 30)_ = 13.68, *p* < 0.01] and aripiprazole [*F*_(3, 32)_ = 9.99, *p* < 0.001]. As this measure was not reduced with PCP alone, this effect was the result of fewer correct responses made in animals that received PCP and cariprazine (0.1 and 0.3 mg/kg, *p* < 0.001 each) or PCP and aripiprazole (all three doses, *p* < 0.05–*p* < 0.001) (Table 2).

For percent omissions, a significant interaction was evident with both cariprazine [*F*_(3, 30)_ = 14.67, *p* < 0.001] and aripiprazole [*F*_(3, 32)_ = 11.49, *p* < 0.001]. No increase was observed in animals treated with PCP alone. Omissions were increased in the groups that received PCP and cariprazine (0.1 or 0.3 mg/kg; *p* < 0.001 each) or PCP and aripiprazole (all three doses, *p* < 0.05 to *p* < 0.001) (Table 2).

**Fig. 3:**
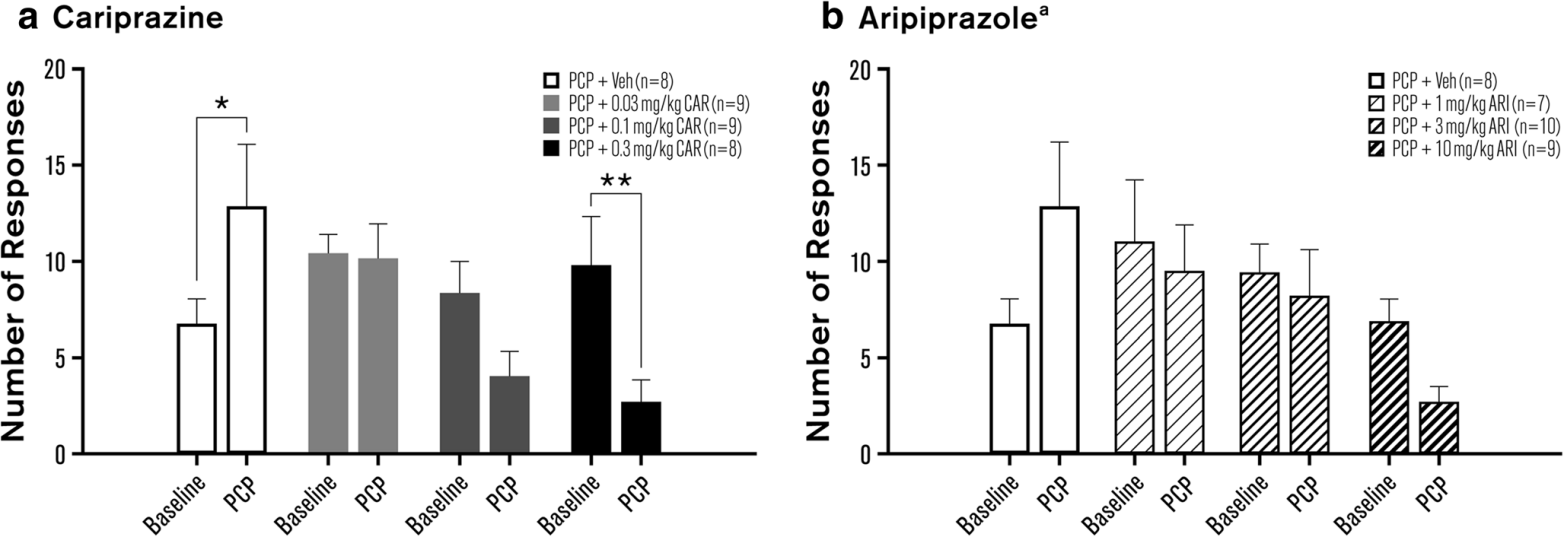
Effects of cariprazine (**a**) and aripiprazole (**b**) on PCP-induced impulsivity: premature responses. Measures are shown as mean ± SEM. *p* values are based on post hoc Fisher least significant difference test (cariprazine) or ANOVA planned comparison tests (aripiprazole). *p < 0.05; **p < 0.01 compared to baseline. ARI aripiprazole, CAR cariprazine, PCP phencyclidine, Veh vehicle. ^a^Two outliers were removed from the PCP + 1 mg/kg aripiprazole group

In the 5-CSRTT, motoric impulsivity is reflected by the number of premature responses before stimulus presentation, although this measure could also reflect timing capabilities (Cope et al. 2016). Premature responding was significantly disrupted with cariprazine [*F*_(3, 30)_ = 6.04, *p* < 0.01] and aripiprazole [*F*_(3, 32)_ = 3.06, *p* < 0.05]. Compared to baseline, premature responding was significantly increased in PCP-treated animals (*p* < 0.05; Fig. 3a, Table 2). No significant difference from baseline was evident in two cariprazine treatment groups (0.03, 0.1 mg/kg). The highest dose of cariprazine (0.3 mg/kg) significantly reduced premature responding compared to baseline performance (*p* < 0.01; Fig. 3a, Table 2). In the aripiprazole experiments, premature responses approached a significant increase in PCP-treated animals (*p* = 0.06) and were decreased in animals treated with PCP and 1 mg/kg aripiprazole (*p* < 0.05; Fig. 3b, Table 2). However, this effect likely reflected the unusually high baseline level of premature responses rather than a reduction per se. Balancing the high level of baseline premature responding in the PCP and 1 mg/kg aripiprazole group (by removing two animals from this group) with that of all other groups at baseline diminished the interaction, and the effect just failed to reach statistical significance [*F*_(3, 30)_ = 2.78, *p* = 0.058]. Aripiprazole attenuated the PCP-induced increase in premature responding at all doses tested (Fig. 3b, Table 2).

**Fig. 4:**
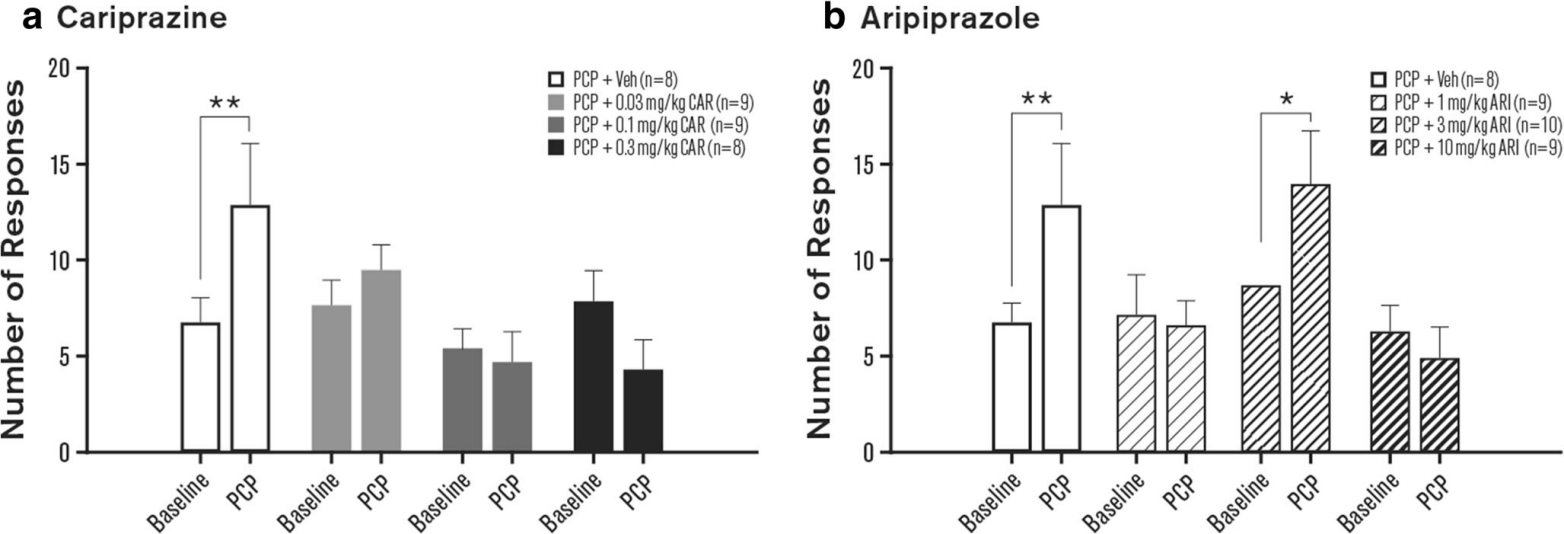
Effects of cariprazine (**a**) and aripiprazole (**b**) on PCP-induced cognitive flexibility: timeout responses. Measures are shown as means ± SEM. *p* values are based on post hoc Fisher least significant difference test. *p < 0.05; **p < 0.01 compared to baseline. ARI aripiprazole, CAR cariprazine, PCP phencyclidine, Veh vehicle

A surrogate measure for cognitive flexibility in the 5-CSRTT is the number of perseverative responses and timeout responses. Cariprazine significantly influenced perseverative responding [*F*_(3, 30)_ = 6.02, *p* < 0.001]. However, this effect was not driven by a PCP-induced increase. Rather, perseverative responding was significantly reduced after cariprazine treatment (0.3 mg/kg, *p* < 0.001; Table 2). No effect on perseverative responding was observed with aripiprazole [*F*_(3, 32)_ = 0.55, ns]. The number of timeout responses was significantly influenced by both cariprazine [*F*_(3, 30)_ = 4.30, *p* < 0.05] and aripiprazole [*F*_(3, 32)_ = 3.37, *p* < 0.05]. Timeout responding was significantly increased in the PCP-treated group compared to baseline (*p* < 0.01); no significant changes from baseline were observed with any cariprazine dose (Fig. 4a, Table 2). In contrast, the PCP-induced increase in timeout responding (*p* < 0.01) was attenuated in animals receiving 1 and 10 mg/kg aripiprazole (Fig. 4b, Table 2). A significant increase in timeout responding was evident in animals receiving PCP and 3 mg/kg aripiprazole (*p* < 0.05; Fig. 4b, Table 2).

When the number of completed trials was analyzed, significant interactions were evident with cariprazine [*F*_(3, 30)_ = 11.59, *p* < 0.001] and aripiprazole [*F*_(3, 32)_ = 7.37, *p* < 0.001]. PCP alone had no effect on trials completed; therefore, the significant interactions were attributable to the significant reduction of the number of trials completed within a session with PCP and cariprazine (0.1 mg/kg, *p* < 0.01; 0.3 mg/kg, *p* < 0.001) or with PCP and aripiprazole (10 mg/kg, *p* < 0.001) (Table 2).

In the 5-CSRTT, a surrogate measure for processing speed can be evaluated by comparing correct latency (latency to correct response) and reward latency (time to reward retrieval). In the cariprazine treatment experiments, no interaction was observed for correct latency [*F*_(3, 30)_ = 2.19, *p* = 0.1], but a main effect of Day was present [*F*_(1, 30)_ = 24.69, *p* < 0.001]. ANOVA did not reveal any effect on reward latency [*F*_(3, 30)_ = 1.01, NS]. Hence, cariprazine treatment slowed processing speed without affecting overall motoric capability. For aripiprazole, a main effect of Day was also evident when correct latency was analyzed [*F*_(1, 32)_ = 12.27, *p* < 0.01], indicating that the latency to make a correct response was elevated in all groups. No significant effect was observed for reward latency, although a main effect of Day approached significance [*F*_(1, 32)_ = 3.66, *p* < 0.1] (Table 2).

## Discussion

The 5-CSRTT assesses a range of cognitive domains, including aspects of sustained attention, motoric impulsivity, speed of processing, and cognitive flexibility. In this study, we have shown that a repeated PCP treatment regimen disrupted several of these domains in rats in ways that are similar to those observed in patients with schizophrenia. Namely, PCP treatment significantly increased the number of incorrect, premature, and timeout responses, which can be considered proxy measurements of attention deficits, motoric impulsivity, and cognitive inflexibility, respectively, symptoms that are frequently observed in patients with schizophrenia (Amitai and Markou 2010). Moreover, these effects occurred without affecting measures such as completed trials or reward latency, suggesting PCP-induced deficits were not the result of non-specific performance alterations. In addition, all three cariprazine doses significantly diminished the PCP-induced increases in incorrect, premature, and timeout responses. However, the two higher cariprazine doses also significantly reduced the number of trials completed, percent accuracy, and number of correct responses, suggesting that these doses resulted in a non-specific suppression of responses. This non-specific response suppression appears to be a characteristic of antipsychotic drugs, as similar non-specific effects due to high doses of antipsychotic compounds were also reported in an earlier study (Amitai et al. 2007). Importantly, the lowest cariprazine dose (0.03 mg/kg) improved the 5-CSRTT performance without inducing these non-specific disruptions, suggesting that this dose is effective in attenuating PCP-induced impairments in cognition. Of note, this cariprazine dose is lower than the antipsychotic-like effective dose (ED_50_ = 0.09 mg/kg) that attenuated PCP-induced increases in locomotor activity in a previous study (Gyertyán et al. 2011). It should be noted, however, that some effects presented here were marginal and only statistically significant when a priori pre-planned comparisons were applied. Nonetheless, the findings of the present study provide support for the hypothesis that low doses of cariprazine may be effective against cognitive symptoms of schizophrenia.

Aripiprazole treatment resulted in a slightly different attention response profile. Two aripiprazole doses (1 and 10 mg/kg) significantly reversed the PCP-induced increases in incorrect and timeout responses. However, all three aripiprazole doses also decreased the percentage of correct responses and increased the number of omissions, suggesting that aripiprazole may directly impair select forms of attention, resulting in a reduction in stimulus detection. While there was an apparent dose-dependent reduction in premature response in aripiprazole-treated animals, the interaction for this measure did not reach statistical significance. While it may be possible that aripiprazole improves PCP-induced increases in motoric impulsivity, further experiments and replication are required before firm conclusions can be made. The effects of drug treatment on 5-CSRTT performance were not evaluated in the absence of PCP; therefore, it is unclear to what extent any or all of these effects of aripiprazole are due to its action on PCP-induced responses vs its direct effects on 5-CSRTT performance, or an interaction between the two. These findings are similar to those of an earlier study, which used the competitive NMDA receptor antagonist CPP to induce cognitive deficits in rats that underwent the 5-CSRTT (Carli et al. 2011). Aripiprazole (1 and 3 mg/kg) ameliorated CPP-induced decreases in accuracy and increases in perseverative overresponding, suggesting that aripiprazole can affect both the attention and the cognitive flexibility domains of cognition at these doses. Furthermore, these data support the doses chosen for the present study, although lower doses could be tested in the future.

Our findings are consistent with those reported in previous studies in which second-generation antipsychotics attenuated PCP- or CPP-induced cognitive deficits in the 5-CSRTT (Amitai et al. 2007; Carli et al. 2011). Clozapine reversed chronic PCP-induced decreases in accuracy and increases in premature responses in the 5-CSRTT, but had no effect on measures of cognitive flexibility (Amitai et al. 2007). In a later study, quetiapine failed to modulate any PCP-induced cognitive deficits modeled by the 5-CSRTT (Amitai and Markou 2009). Olanzapine (0.3 and 1.0 mg/kg) reduced CPP-induced attention deficits and premature responding, but did not affect perseverative responses (Carli et al. 2011). Taken together, these studies indicate that different antipsychotics can specifically modulate discrete cognitive domains. Clinical studies have also shown that different antipsychotics are effective against different aspects of cognition in patients with schizophrenia (Meltzer and McGurk 1999; Sharma and Mockler 1998; Strous et al. 2006; Velligan et al. 2002). At the dose that specifically attenuated PCP-induced cognitive deficits, cariprazine exerted broad effects on multiple cognitive domains, suggesting that cariprazine may be of use in patients with schizophrenia who exhibit a range of cognitive symptoms.

The diverse effects of different antipsychotic compounds could be related to their varied receptor pharmacology profiles. Clozapine, quetiapine, and olanzapine are dopamine D_2_ receptor and serotonin 5-HT_2A_ receptor antagonists (Bymaster et al. 1996; Ellenbroek and Cesura 2015; Moore et al. 1992; Schmidt et al. 2001). In contrast, cariprazine and aripiprazole are partial agonists at dopamine D_2_ and D_3_ receptors and serotonin 5-HT_1A_ receptors and antagonists at 5-HT_2A_ receptors (Kiss et al. 2010). Importantly, alterations in glutamate transmission can impact dopamine levels, as NMDA receptor antagonism results in aberrant dopamine transmission (Adams et al. 2002; Kapur and Seeman 2002; Pouvreau et al. 2016). Moreover, NMDA antagonism induces upregulation of striatal dopamine D_2_ receptors (Nair et al. 1998). Striatal dopamine D_2_ receptor overexpression produces motivational and cognitive impairment associated with schizophrenia (Kellendonk et al. 2006; Simpson et al. 2011) and alterations in the PFC inhibitory transmission (Li et al. 2011) reminiscent to those observed after the PCP treatment. Increased expression of striatal dopamine D_2_ receptors may, therefore, contribute to the disruptive effects of NMDA antagonists on attentional processes and provide a potential mechanism for the behavioral attenuation when compounds that modulate the activity of dopamine D_2_-like receptors are administered. While dopamine D_2_ receptors are expressed throughout the brain, high dopamine D_3_ receptor expression occurs in several particular regions of the brain, including the prefrontal cortex, nucleus accumbens, and ventral tegmental area, that are associated with negative, cognitive, and mood symptoms of schizophrenia (Gross and Drescher 2012). Because the compounds in this study were administered systemically, it is not possible to pinpoint the exact region of the brain in which they function to modulate cognitive impairment. However, an impaired cognition associated with schizophrenia is thought to be associated with hypofunction of the prefrontal cortex, including reduced dopaminergic activity (Goldman-Rakic et al. 2004; Slifstein et al. 2015).

The fact that cariprazine acts as a partial agonist at D_3_ and D_2_ receptors may have important implications for its therapeutic profile that makes its use preferable to that of pure D_3_/D_2_ receptor antagonists. It has been hypothesized that a general dysregulation of dopaminergic systems in patients with schizophrenia can result in both *hyperactivity* in circuits associated with positive symptoms and *hypoactivity* in those associated with cognitive and negative symptoms (Maia and Frank 2017). Partial agonists, by definition, have the ability to constrain activity in neurotransmitter-receptor circuits within a certain range, potentially allowing for the normalization of both hyperactive and hypoactive circuits. Therefore, the partial agonist properties of cariprazine may allow for the simultaneous treatment of the multiple symptom domains of schizophrenia, acting as an antagonist to reduce dopaminergic hyperactivity associated with positive symptoms and as an agonist to increase dopaminergic activity in brain regions associated with negative and cognitive symptoms.

Based on the preferential distribution of D_3_ receptors in corticolimbic circuits (Sokoloff et al. 1990), it has been suggested that dopamine antagonists displaying high affinity for both D_3_ and D_2_ receptors may yield a favorable antipsychotic therapeutic profile in terms of maximizing efficacy (Gyertyán et al. 2008; Kiss et al. 2008). Cariprazine exhibits such a binding profile, having a higher affinity and selectivity for the D_3_ vs the D_2_ receptor. In contrast, aripiprazole has a higher affinity and selectivity for the D_2_ vs the D_3_ receptor, as measured in various in vitro assays (de Bartolomeis et al. 2015; Kiss et al. 2010). Moreover, in the in vivo rodent studies, cariprazine but not aripiprazole showed high occupancy of both D_2_ and D_3_ receptors at antipsychotic-like effective doses (Gyertyán et al. 2011), indicating that potentially, cariprazine would have a greater impact on D_3_ receptor activity than aripiprazole. Interestingly, while the low and high doses of aripiprazole attenuated PCP-induced impairments in incorrect responding, the medium dose was not effective. Dopamine transmission-mediated modulation of cognitive processing is extremely sensitive to an optimal range of activity (Floresco 2013). While further investigations are necessary to elucidate the precise mechanism underlying this unusual effect, it may reflect aripiprazole displaying optimal or sub-optimal effects on dopamine transmission depending on the dose administered. The absence of this usual effect in cariprazine treated rats may also reflect differences in D_2_ and D_3_ receptor selectivity, but the precise mechanism for this dissociation is unknown and requires further investigation. Cariprazine was previously shown to reverse behavioral, cognitive, and social deficits in PCP-induced animal models of schizophrenia (Gyertyán et al. 2011; Neill et al. 2016; Watson et al. 2016; Zimnisky et al. 2013). These effects are likely due to cariprazine’s partial agonist activity on the D_3_ receptor, as cariprazine significantly ameliorated cognitive deficits induced by acute administration of PCP in wild-type mice, but not in D_3_ receptor knockout mice (Zimnisky et al. 2013). The more specific effect of cariprazine compared with aripiprazole on impaired cognition induced by PCP in this study may therefore be due to its preferential activity at the D_3_ receptor. Future studies using dopamine D_3_ receptor knockout mice in the 5-CSRTT are necessary to determine whether the effects of cariprazine are specific to the D_3_ receptor.

In summary, cariprazine exhibited a better overall cognitive profile than aripiprazole in the attenuation of PCP-induced deficits in the 5-CSRTT performance. Although higher tested doses of both compounds appeared to induce non-specific effects, the lowest dose of cariprazine significantly reversed the inappropriate responses induced by PCP and may therefore have potential for improving attentional impairment and other cognitive defects associated with schizophrenia.
