# Correction to: The Effects of Cariprazine and Aripiprazole on PCP-Induced Deficits on Attention Assessed in the 5-Choice Serial Reaction Time Task

**DOI:** 10.1007/s00213-018-4884-x

**Published:** 2018-03-21

**Authors:** Samuel A. Barnes, Jared W. Young, Athina Markou, Nika Adham, István Gyertyán, Béla Kiss

**Affiliations:** 1 Department of Psychiatry, School of Medicine, University of California San Diego, 9500 Gilman Drive, M/C 0603, Room BSB2202, La Jolla, CA 92093 USA; 2 Allergan, Jersey City, NJ USA; 3 MTA-SE NAP B Cognitive Translational Behavioral Pharmacology Group, Department of Pharmacology and Pharmacotherapy, Semmelweis University, Budapest, Hungary; 4 Institute of Cognitive Neuroscience and Psychology, Research Center for Natural Sciences, MTA, Budapest, Hungary; 5 Gedeon Richter Plc, Budapest, Hungary

**Correction to: Psychopharmacology**

**https://doi.org/10.1007/s00213-018-4857-0**

The article The Effects of Cariprazine and Aripiprazole on PCP-Induced Deficits on Attention Assessed in the 5-Choice Serial Reaction Time Task, written by Samuel A. Barnes, Jared W. Young, Athina Markou, Nika Adham, István Gyertyán, Béla Kiss, was originally published electronically on the publisher’s internet portal (currently SpringerLink) on 22 February 2018 without open access.

With the author(s)’ decision to opt for Open Choice the copyright of the article changed on 19 March 2018 to © The Author(s) 2018 and the article is forthwith distributed under the terms of the Creative Commons Attribution 4.0 International License (http://creativecommons.org/licenses/by/4.0/), which permits use, duplication, adaptation, distribution and reproduction in any medium or format, as long as you give appropriate credit to the original author(s) and the source, provide a link to the Creative Commons license and indicate if changes were made.

